# Supplementary material for: Laboratory evaluation of open source and commercial electrical conductivity sensor precision and accuracy: How do they compare?
Source: PLoS One. 2023 May 4;18(5):e0285092. doi: 10.1371/journal.pone.0285092 (PMC10159144; doi:10.1371/journal.pone.0285092)
Supplement: Attachment — Submitted filename: Response to Reviewers.docx [file pone.0285092.s002.docx]

Response to Reviewers

“Laboratory evaluation of open source and commercial electrical conductivity sensor precision and accuracy: How do they compare?” (PONE-D-22-14296)

**General Comments from PLOS ONE Editor**

Comment 1: If applicable, we recommend that you deposit your laboratory protocols in protocols.io to enhance the reproducibility of your results. Protocols.io assigns your protocol its own identifier (DOI) so that it can be cited independently in the future. For instructions see: <https://journals.plos.org/plosone/s/submission-guidelines#loc-laboratory-protocols>. Additionally, PLOS ONE offers an option for publishing peer-reviewed Lab Protocol articles, which describe protocols hosted on protocols.io. Read more information on sharing protocols at <https://plos.org/protocols?utm_medium=editorial-email&utm_source=authorletters&utm_campaign=protocols>.

Response 1: We have provided the user manuals and/or instructions for all sensor calibrations in the data package. See new “Data and code availability” statement starting on line 423.

**Journal Requirements to be addressed/double-checked**

Comment 2: Please **ensure that your manuscript meets PLOS ONE's style requirements, including those for file naming**. The PLOS ONE style templates can be found at <https://journals.plos.org/plosone/s/file?id=wjVg/PLOSOne_formatting_sample_main_body.pdf> and <https://journals.plos.org/plosone/s/file?id=ba62/PLOSOne_formatting_sample_title_authors_affiliations.pdf>

Response 2: We have reviewed the manuscript for adherence to PLOS ONE’s style requirements, including those for file naming, and made all necessary changes to the manuscript style and file naming conventions.

Comment 3: In your Data Availability statement, **you** **have not specified where the minimal data set underlying the results described in your manuscript can be found**. PLOS defines a study's minimal data set as the underlying data used to reach the conclusions drawn in the manuscript and any additional data required to replicate the reported study findings in their entirety. All PLOS journals require that the minimal data set be made fully available. For more information about our data policy, please see <http://journals.plos.org/plosone/s/data-availability>.

Response 3: We have added a “Data and code availability” statement starting on line 4235 specifying where the minimal data set underlying the results described in our manuscript can be found. We have also included the data set as Table S1 in the new Supplemental Information section.

Comment 4: Please **review your reference list to ensure that it is complete and correct**. If you have cited papers that have been retracted, please include the rationale for doing so in the manuscript text, or remove these references and replace them with relevant current references. Any changes to the reference list should be mentioned in the rebuttal letter that accompanies your revised manuscript. If you need to cite a retracted article, indicate the article’s retracted status in the References list and also include a citation and full reference for the retraction notice.

Response 4: We have double-checked the reference list to ensure that it is complete and correct. No article retractions were necessary. We added the following 11 new references, including one new reference [35] on using EC to investigate groundwater-surface water mixing and 10 new references in response to comments from the Reviewers [46, 47, 48, 49, 50, 51, 52, 53, 56, 61].

[35]	Cox MH, Su GW, Constantz J. Heat, chloride, and specific conductance as ground water tracers near streams. Ground Water. 2007;45(2):187-95.

[46]	Lockridge G, Dzwonkowski B, Nelson R, Powers S. Development of a Low-Cost Arduino-Based Sonde for Coastal Applications. Sensors. 2016;16(4):528.

[47]	Gillett D, Marchiori A. A Low-Cost Continuous Turbidity Monitor. Sensors. 2019;19(14):3039.

[48]	Kelley CD, Krolick A, Brunner L, Burklund A, Kahn D, Ball WP, et al. An affordable open-source turbidimeter. Sensors (Basel). 2014;14(4):7142-55.

[49]	Murphy K, Heery B, Sullivan T, Zhang D, Paludetti L, Lau KT, et al. A low-cost autonomous optical sensor for water quality monitoring. Talanta. 2015;132:520-7.

[50]	Murphy K, Sullivan T, Heery B, Regan F. Data analysis from a low-cost optical sensor for continuous marine monitoring. Sensors and Actuators B: Chemical. 2015;214:211-7.

[51]	Fulton SG. Engineering Hydrologic Flowpaths for Water Quality During Mountaintop Removal and Valley Fill Mining [Dissertation]. Ann Arbor, MI: University of Georgia; 2020.

[52]	Atlas Scientific Environmental Robotics. EZO Conductivity Accuracy Graph 2022 March 12, 2023. Available from: <https://files.atlas-scientific.com/conductivity_accuracy_graph.pdf>.

[53]	Atlas Scientific Environmental Robotics. Arduino Mega Conductivity Sample Code 2022 March 12, 2023. Available from: <https://files.atlas-scientific.com/Arduino-Mega-EC-Sample-Code.pdf>.

[56]	YSI Incorporated. Professional Plus Calibration Tips (Rev. A) 2010. Available from: <https://www.ysi.com/ysi-blog/water-blogged-blog/2018/06/ysi-prodss-water-quality-meter-calibration-tips-the-ultimate-guide>.

[61]	USGS. Chapter A6.3. Specific Conductance. Report. Reston, VA; 2019. Report No.: 9-A6.3.

**Review Comments to the Author**

***Reviewer #1***

Major Comments

Comment 1: Provide on a GitHub or other repository the details of how to operate and log the Atlas sensor (or provide links to other who have done it).

Response 1: We added the following information and an additional citation [53] to the manuscript to address this issue:

1) Additional text in the manuscript (see paragraph starting line 212) briefly describing the Arduino IDE source code (ino file) [53] that we modified to operate the Atlas EC sensor.
2) We added a “Data and code availability” statement describing the data repository where we will publish all data and statistical analysis code underlying the findings in our study along with the .ino file and instructions to operate the OS Atlas EC sensor, as well as the user manuals to operate the YSI EcoSense® EC300A and Decagon CTD-10 commercial sensors. The data package will be published at the U.S. Department of Energy’s Environmental System Science Data Infrastructure for a Virtual Ecosystem (ESS-DIVE) repository (<https://ess-dive.lbl.gov/about/>). The persistent doi for the repository will be provided once the data package is approved for publication and will be available prior to manuscript publication.

[53]	Atlas Scientific Environmental Robotics. Arduino Mega Conductivity Sample Code 2022 March 12, 2023. Available from: <https://files.atlas-scientific.com/Arduino-Mega-EC-Sample-Code.pdf>.

Comment 2: 2pt calibration, while they may be industry standard are problematic as between every two points passes a single line and hence the calibration process itself is not useful in providing likely uncertainties. It would be useful if you could discuss this issue.

Response 2: To support our use of a 2-point calibration, we revised the paragraph starting on line 182 describing the OS Atlas EC probe kit, including a new citation [52], to provide additional information from the manufacturer describing the linear response of the sensor over the range of measurements used for this study and the resultant sensor accuracy for 2-point calibrations. We also included a new citation for a related study [51], of which this study was a part and revised the paragraph starting on line 227 in the Results and Discussion section describing when to use 2-point or 3-point calibrations, including a new supporting citation [56]. Citations added to address this concern are listed below:

[51]	Fulton SG. Engineering Hydrologic Flowpaths for Water Quality During Mountaintop Removal and Valley Fill Mining [Dissertation]. Ann Arbor, MI: University of Georgia; 2020.

[52]	Atlas Scientific Environmental Robotics. EZO Conductivity Accuracy Graph 2022 March 12, 2023. Available from: <https://files.atlas-scientific.com/conductivity_accuracy_graph.pdf>.

[56]	YSI Incorporated. Professional Plus Calibration Tips (Rev. A) 2010. Available from: <https://www.ysi.com/ysi-blog/water-blogged-blog/2018/06/ysi-prodss-water-quality-meter-calibration-tips-the-ultimate-guide>.

Comment 3: It is not clear why you mention the DO and pH evaluations of the Atlas. How are they relevant here?

Response 3: We included experimental results cited by authors on the (high) accuracy and precision of other OS Atlas sensors besides EC (e.g., DO, pH, temperature) to present a more complete description of investigations on Atlas sensor data quality. This additional information on data quality for pH and DO sensors was presented primarily for discussion purposes. However, we also believe that providing additional information on the overall high data quality for non-EC sensors made by Atlas (or any other OS sensor manufacturer) is not only an important contribution to the relatively small body of literature currently available on OS sensor data quality, but it may also be helpful to other researchers to learn that Atlas, or any other vendor, manufactures several different types of sensors. We have revised the paragraph starting on line 287 to clarify that relatively consistent results across studies show that Atlas sensors in general perform as well as or better than commercial sensors.

Comment 4: mg/l are not appropriate salinity units (l. 320).

Response 4: We agree, the appropriate units for salinity are typically expressed in parts per thousand (ppt) or practical salinity units (PSU). We revised the text to include the appropriate units as follows: “(OS Atlas EC voltage measurements converted to parts per thousand [ppt] or practical salinity units [PSU] using the EZO EC circuit)” (see lines 368-369).

Comment 5: The comparison is not strictly fair as you do not calibrate the Decagon sensor. Why not mimic the calibration procedure by forcing the Decagon to agree with the two points (e.g., using a linear transformation) and then evaluated [stet] how well it does?

Response 5: We have revised the paragraph starting on line 227 to explain why we did not calibrate the Decagon CTD-10. In short, like many other commercial sensors, users must rely on factory calibrations because the CTD-10 sensor has an embedded chip that uses proprietary software that is inaccessible to users to perform calibrations (specifically, see lines 242-244). However, we recognize that it is critical to periodically check sensor calibrations both in-house and in the field, particularly during longer field deployments, to check for sensor drift. If the CTD-10 has drifted, however, the sensor must be returned to Meter Group for a calibration check and recalibration or is replaced if recalibration cannot be performed to Meter’s specification.

Comment 6: A very important point you do not address is sensor drift. How long after a calibration can we expect it to remain the same? If the Atlas drift much faster than the Decagon that is something your reader will want to know. If you cannot evaluate it with your data to date, at least mention it in the discussion.

Response 6: We have revised the paragraph starting on line 4I have02 in the Conclusions section to address the issue of calibration drift and include the following new supporting citation [61]:

[61]	USGS. Chapter A6.3. Specific Conductance. Report. Reston, VA; 2019. Report No.: 9-A6.3.

Comment 7: Please provide an explanation of how your evaluation is different from that of (50).

Response 7: We revised the paragraph starting on line 287 explaining the major methodological and technological differences between our study and that of Mendez-Barroso et al. (2020) [60].

Minor Comments

Comment 8: l. 182: 'that' should be 'than'.

Response 8: “That” has been changed to “than.”

Comment 9: Fi.g [stet] 3. and 5: 'OS/C' is smooshed.

Response 9: Fig 3 and Fig 5 have been corrected to remove “smooshed” text.

***Reviewer #2***

Major Comments (“…only to consider and address”)

Comment 1: I am wondering why the study only consider one type of OS EC sensor. I think the aim of this paper is also to evaluate the performance of different OS EC units, and I believe there are many available designs out there on the market with a cost ranging between $10 and $400.

Response 1: The purpose of this study was to assess the laboratory performance (i.e., sensor accuracy and precision) of Atlas EC and Decagon CTD-10 sensors that were used in a related study as part of Dr. Fulton’s dissertation research on the effects of experimental mine reclamation practices titled “Engineering Hydrologic Flowpaths for Water Quality During Mountaintop Removal and Valley Fill Mining (see line 132 for new citation added [51]). When we began developing an OS wireless sensor network for that study in 2015, there were far fewer low-cost, off-the-shelf, user-friendly OS EC sensors available than are available today. To address your concern, we explain the change in the market availability for OS EC sensors in the first paragraph of the Conclusions section starting on line 377 and provide two examples of less expensive OS EC sensors.

[51]	Fulton SG. Engineering Hydrologic Flowpaths for Water Quality During Mountaintop Removal and Valley Fill Mining [Dissertation]. Ann Arbor, MI: University of Georgia; 2020.

Comment 2: I do not think data logger will become a source of error no matter you use Arduino or commercial products. As long as you make the logger support the standard data communication [stet] protocal [stet] (e.g., serial communication, SDI-12), not sure why the data logger might not correctly handle the transmission.

Response 2: Point well taken. As described above in Response 1, this study was a component of Dr. Fulton’s dissertation research, which involved building and deploying an OS wireless sensor network to remotely monitor mine effluent specific conductance and streamflow. We were simply being prudent in the event of future sensor or wireless sensor network failures during remote deployments, and wanted to ensure the OS data logger successfully collected data via the OS/C Decagon EC sensor. We have revised the paragraph starting on line 143 to better explain our rationale for evaluating the OS/C Decagon configuration.

Minor comments

Comment 3: Line 116-119: citations needed

Response 3: The following five new citations have been added (see paragraph starting on line 117) to include papers that evaluated non-EC OS sensor performance against commercial EC sensors:

[46]	Lockridge G, Dzwonkowski B, Nelson R, Powers S. Development of a Low-Cost Arduino-Based Sonde for Coastal Applications. Sensors. 2016;16(4):528.

[47]	Gillett D, Marchiori A. A Low-Cost Continuous Turbidity Monitor. Sensors. 2019;19(14):3039.

[48]	Kelley CD, Krolick A, Brunner L, Burklund A, Kahn D, Ball WP, et al. An affordable open-source turbidimeter. Sensors (Basel). 2014;14(4):7142-55.

[49]	Murphy K, Heery B, Sullivan T, Zhang D, Paludetti L, Lau KT, et al. A low-cost autonomous optical sensor for water quality monitoring. Talanta. 2015;132:520-7.

[50]	Murphy K, Sullivan T, Heery B, Regan F. Data analysis from a low-cost optical sensor for continuous marine monitoring. Sensors and Actuators B: Chemical. 2015;214:211-7.

Comment 4: Line 249-265: what’s the purpose of this paragraph? You are talking about EC sensor, why need to mention the pH and DO sensor from the same company?

Response 4: Reviewer #1 asked a similar question, which we responded to and will provide the same response here. We included experimental results cited by authors on the (high) accuracy and precision of other OS Atlas sensors besides EC (e.g., DO, pH, temperature) to present a more complete description of authors' investigations on Atlas sensor data quality. This additional information on data quality for pH and DO sensors was presented primarily for discussion purposes. However, we also believe that providing additional information on the overall high data quality for non-EC sensors made by Atlas (or any other OS sensor manufacturer) is not only an important contribution to the relatively small body of literature currently available on OS sensor data quality, but it may also be helpful to other researchers to learn that Atlas, or any other vendor, manufactures several different types of sensors. We have revised the paragraph starting on line 287 to clarify that relatively consistent results across studies show that Atlas sensors in general perform as well as or better than commercial sensors.
